# Detection of abnormal extraocular muscles in small datasets of computed tomography images using a three-dimensional variational autoencoder

**DOI:** 10.1038/s41598-023-28082-5

**Published:** 2023-01-31

**Authors:** Yeon Woong Chung, In Young Choi

**Affiliations:** 1 Department of Ophthalmology and Visual Science, St. Vincent’s Hospital, College of Medicine, The Catholic University of Korea, Seoul, Republic of Korea; 2 Department of Medical Informatics, College of Medicine, The Catholic University of Korea, Banpo Dae-Ro 222, Seoul, 06591 Republic of Korea

**Keywords:** Computer science, Eye diseases, Outcomes research

## Abstract

We sought to establish an unsupervised algorithm with a three–dimensional (3D) variational autoencoder model (VAE) for the detection of abnormal extraocular muscles in small datasets of orbital computed tomography (CT) images. 334 CT images of normal orbits and 96 of abnormal orbits diagnosed as thyroid eye disease were used for training and validation; 24 normal and 11 abnormal orbits were used for the test. A 3D VAE was developed and trained. All images were preprocessed to emphasize extraocular muscles and to suppress background noise (e.g., high signal intensity from bones). The optimal cut-off value was identified through receiver operating characteristic (ROC) curve analysis. The ability of the model to detect muscles of abnormal size was assessed by visualization. The model achieved a sensitivity of 79.2%, specificity of 72.7%, accuracy of 77.1%, F1-score of 0.667, and AUROC of 0.801. Abnormal CT images correctly identified by the model showed differences in the reconstruction of extraocular muscles. The proposed model showed potential to detect abnormalities in extraocular muscles using a small dataset, similar to the diagnostic approach used by physicians. Unsupervised learning could serve as an alternative detection method for medical imaging studies in which annotation is difficult or impossible to perform.

## Introduction

With the increasing availability of large-scale medical research datasets, deep neural networks are now being widely used for medical image analysis^1^. Large datasets have led to considerable advancements in deep neural networks^2^. The ImageNet dataset, an open access large-scale datasets, is used as the standard for image classification^3–5^. Compared with images generally available in the public domain, medical images have two main disadvantages for deep learning (DL). First, it is difficult to collect sufficient medical image datasets. Second, few annotated medical datasets are available because they require a laborious annotation process to be performed by the trained experts and are restricted by legal issues associated with public access to private medical information^6,7^. Because supervised DL generally relies on large amounts of data that have been accurately annotated by experts, the data preparation phase of medical imaging studies can be challenging. Additionally, clinical manifestations that require diagnosis based on imaging studies (e.g., computed tomography (CT) or magnetic resonance imaging (MRI)) differ in size, shape, and location, even among patients with the same diagnosis. Therefore, it is difficult for supervised DL to clearly identify specific patterns of the disease.

In contrast to supervised DL, unsupervised DL does not require annotated data. Unsupervised DL exhibits self-organization that captures patterns as combinations of neural feature preferences without annotation. A primary application of unsupervised DL is anomaly detection, which involves the identification of rare items, events, or observations that are clearly different from normal data. This identification is possible because unsupervised DL learns normal features through the compression and reconstruction of normal data^8^. This approach can serve as an alternative to supervised DL for the detection of diseases with manifestations varying in size, shape, and location, including rare diseases. Furthermore, anomaly detection techniques can reduce the need for annotation, which is required for supervised DL (particularly in medical imaging studies); they can also reduce the interobserver bias that occurs among experts who perform the annotation process.

**Figure 1:**
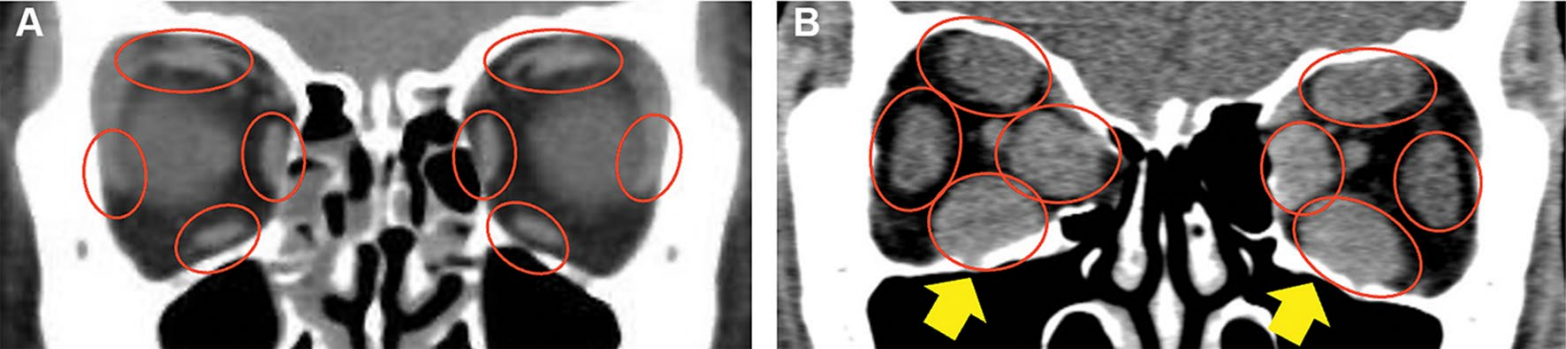
Representative images of extraocular muscles in normal (**A**) and TED (**B**). Red hollow circles indicate extraocular muscles in the human orbit. (**A**) Normal extraocular muscles shown in the coronal view in orbit CT are relatively flat. (**B**) Extraocular muscles in TED are thick and clubby compared with normal extraocular muscles, which are called extraocular muscle enlargements**.** In general, extraocular muscle enlargements in TED start at the inferior rectus muscles (yellow arrows).

The orbit is a small and complex space that contains many important structures, including the eyeball, nerves required for vision and eyeball movements, extraocular muscles, blood vessels, and supporting tissues. Among these intraorbital structures, the extraocular muscles only account for 6.5 cm^3^ of the total volume of the bony orbit (30.1 cm^3^)^9^. Thyroid eye disease (TED), also known as thyroid-associated ophthalmopathy, is the most frequent extrathyroidal manifestation of hyperthyroidism; it causes increases in the volumes of extraocular muscles, orbital connective tissue, and adipose tissue^10^. The diagnosis of TED is based on thyroid function laboratory parameters (T3, free T4, and thyroid-stimulating hormone) and the identification of extraocular muscle enlargement (particularly involving the inferior rectus muscle) via medical imaging studies, such as orbital CT and MRI. Figure 1 shows differences in extraocular muscles between normal and TED. However, in general, the size and shape of extraocular muscles in TED are less prominent than in the images shown in Fig. 1, and sometimes even radiologic experts cannot distinguish muscle enlargements in TED. Despite attempts to establish normative measurements^11–13^, the assessment of muscle enlargement is often subjective and requires comparison with the opposite orbit or prior qualitative experience^14^. DL may be useful for the diagnosis of TED because it is generally superior to humans in terms of calculating and comparing size or volume between normal extraocular muscles and those of TED, especially when the difference is less prominent if a model is appropriately trained. However, because the extraocular muscles are thin and long, it is challenging for human experts to accurately annotate them for supervised DL. Unsupervised DL offers an alternate method for discriminating abnormal and normal extraocular muscles if features of the latter can be efficiently extracted.

In this paper, we propose an unsupervised learning model with three–dimensional (3D) convolutional neural network structures for the detection of abnormal extraocular muscles in orbital CT images of patients with TED. We evaluate the usefulness of this model for detecting small abnormal structures that are difficult to annotate in a small medical image datasets.

## Results

**Figure 2:**
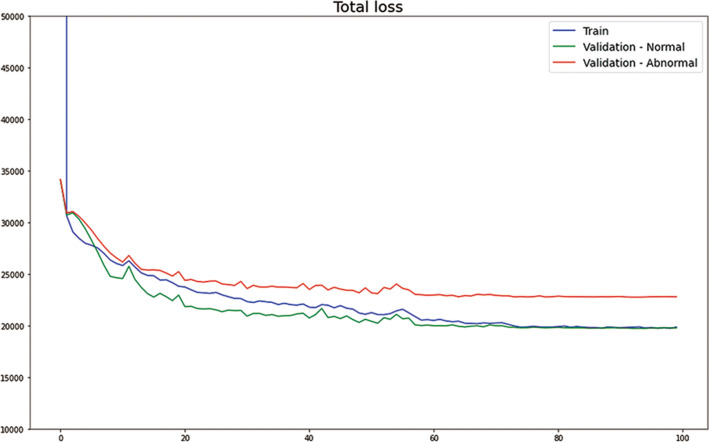
Total losses according to training epoch. Blue line: normal training dataset, green line: normal validation CT dataset, red line: abnormal validation CT dataset. No validation data were used during model training.

The model had stable training and validation curves. The training and both validation curves showed that the model did not overfit the training data. During a few training epochs, the normal and abnormal validation CT groups were not clearly distinguished. However, the two validation CT groups differed in terms of mean total loss after 10 epochs, and this difference was maintained after 20 epochs (Fig. 2).

**Figure 3:**
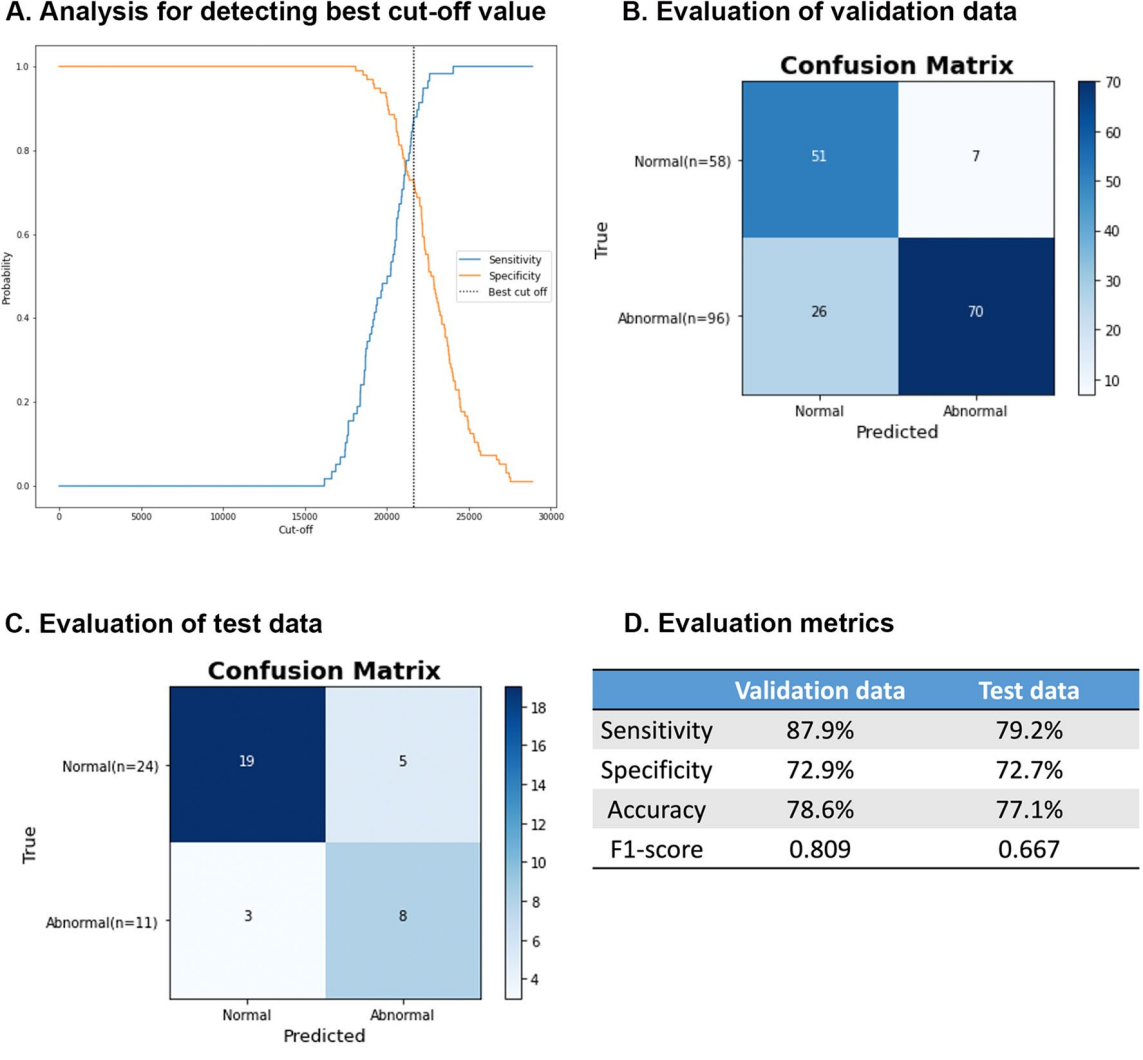
(**A**) Analysis for determining the best cut-off value using receiver operating characteristic (ROC) curve analysis. The best cut-off value was 21,636. (**B**) Confusion matrix between true and predicted validation CT images according to the best cut-off value. (**C**) Confusion matrix between true and predicted test CT images using the same best cut-off value. (**D**) The sensitivity, specificity, accuracy, and F1-score values of validation and test data were presented.

From the ROC curve analysis, the best cut-off value was identified based on the total losses of the model (Fig. 3A). The confusion matrix of validation dataset is shown in Fig. 3B according to the best cut-off value. It demonstrated a sensitivity (the accuracy of normal CT group) of 87.9%, specificity (the accuracy of abnormal CT group) of 72.9%, accuracy of 78.6%, and F1–score of 0.809 (Fig. 3D). Figure 3C shows the confusion matrix of test dataset according to the same best cut-off value. It achieved a sensitivity of 79.2%, specificity of 72.7%, accuracy of 77.1%, F1–score of 0.667 (Fig. 3D), and area under the ROC of 0.801.

**Figure 4:**
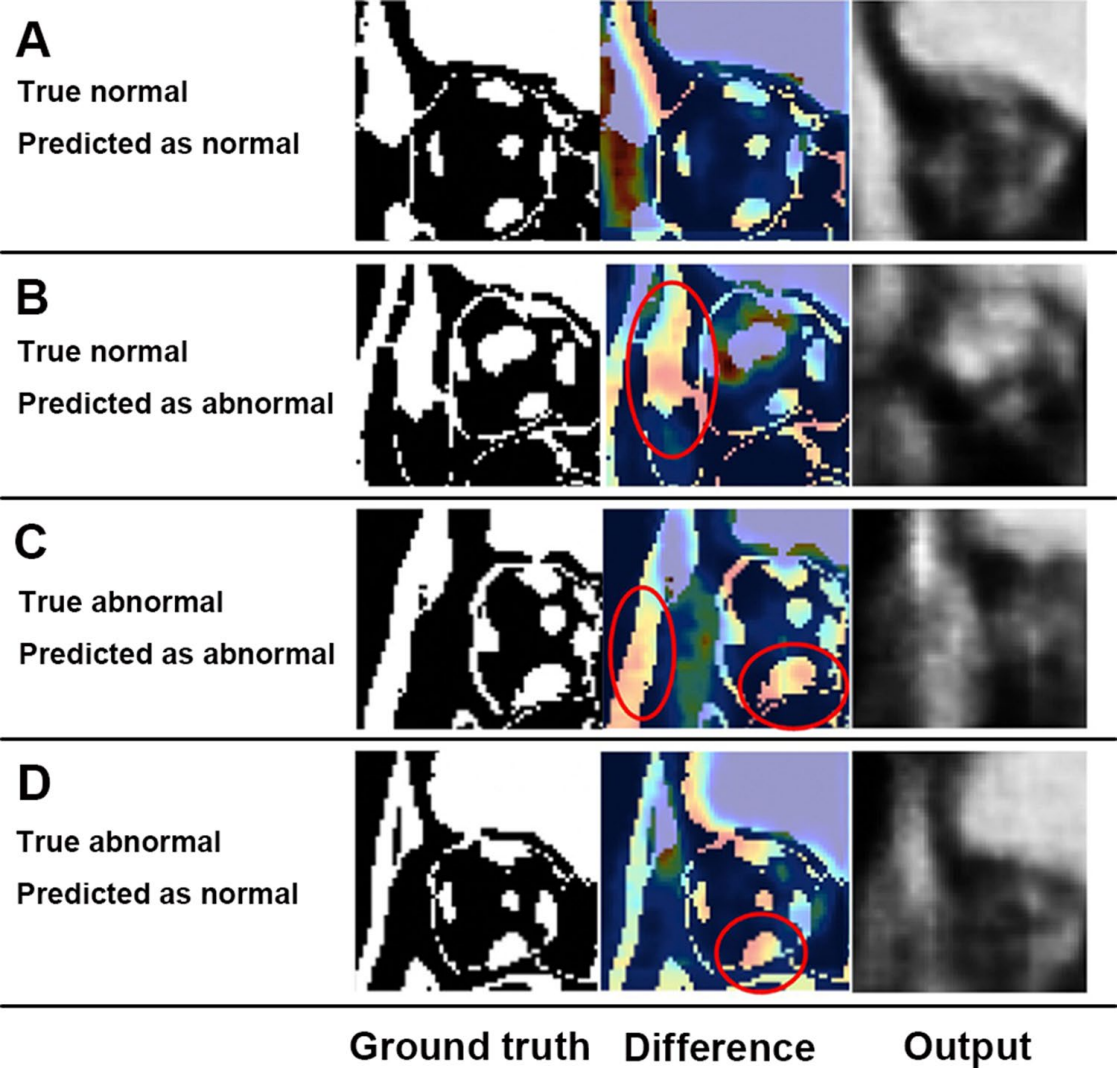
(**A**) Representative normal CT images correctly identified by the model. (**B**) Representative presentation of normal CT images erroneously identified as abnormal. Differences between input and output images are also seen in extraorbital structures (red hollow circles). (**C**) Representative presentation of abnormal CT images correctly identified by the model. Differences between input and output images present in the inferior rectus muscle and extraorbital structures (red hollow circles). (**D**) Representative presentation of abnormal CT images erroneously identified as normal. Differences between input and output images are mainly present in the inferior rectus muscle. However, the total loss values of these images did not exceed the best cut-off value. The ground truth images (left) represent input images. The output images (right) represent images passed through the 3D VAE model. The difference map shows pixel-wise squared difference between input and output images. A large difference is represented by red color.

Normal CT images correctly identified by the model exhibited good reconstruction of extraocular muscles, although there were some differences in corresponding pixel positions between the input and output images (Fig. 4A). However, normal CT images erroneously classified as abnormal showed large differences in extraorbital structures between the input and output images (Fig. 4B).

Abnormal CT images correctly identified by the model showed large differences in the extraocular muscles, while some abnormal CT images also showed large differences in extraorbital structures (Fig. 4C). Additionally, abnormal CT images erroneously identified as normal showed differences in extraocular muscle reconstruction, but the total loss did not exceed the best cut-off value (Fig. 4D).

## Discussion

To our knowledge, this study is the first ophthalmology study to use unsupervised DL to detect abnormalities in small human structures (extraocular muscles) in a small dataset of orbital CT images. The results showed that unsupervised DL may be a useful alternative when annotations are difficult or impossible to perform.

In the literature, the brain is the main organ assessed by unsupervised DL. One study exhaustively compared AE-based methods for anomaly segmentation in brain MRI, which relies on the modeling of healthy anatomy to detect abnormal structures^8^. In another study, an unsupervised medical anomaly detection generative adversarial network reliably detected the accumulation of subtle anatomical abnormalities and hyperintense enhancing lesions^15^. Notably, these studies attempted to detect abnormalities via DL using only normal medical images.

In an ophthalmology study, an anomaly detection model was used to identify signs of ocular diseases in color retinal fundus images^16^. A generative adversarial network model was developed and evaluated using 90,499 retinal fundus images, and an area under the ROP curve value of 0.896, sensitivity of 82.69%, and specificity of 82.63% for detecting abnormal fundus images were achieved. In the present study, we achieved an area under the ROC curve value of 0.801, sensitivity of 79.2%, specificity of 72.7% with test dataset. There are two main differences between the present study and the previous one using retinal fundus images. First, fewer datasets were used in our study. Compared with fundus images, it is more difficult to obtain a large clinical dataset that consists only of normal CT images, because imaging studies (e.g., CT or MRI) are only performed when an ophthalmologist suspects an extraocular anatomical abnormality based on the patient’s history and physical examination results. In contrast, fundus images are routinely acquired for differential diagnosis when a patient exhibit reduced vision. Thus, a considerable difference in the numbers of fundus and orbital CT images in a single medical center should be expected. A larger orbital CT dataset may decrease the total loss caused by extraorbital structures shown in Fig. 4B and improve the performance of our 3D VAE model. Second, the difference between 2 and 3D images has led to a difference in performance. Fundus images represent two dimensions, whereas CT images represent three dimensions. Thus, additional parameters are needed when training a DL model focused on 3D structures, compared with a DL model focused on 2D structures; DL models focused on 3D structures are more complex and sensitive to data scale and hyperparameters^17^.

Bengs et al. evaluated and compared 2D and 3D VAE directly for unsupervised anomaly segmentation in brain MRI^18^. They investigated whether using increased spatial context by using MRI volumes combined with spatial erasing leads to improved unsupervised anomaly segmentation performance compared to learning from slices. As a result, they reported that the 3D VAE outperformed the 2D VAE and had the advantage of volumetric context. In medical imaging studies such as CT or MRI, 3D volumetric context is important in the diagnosis and interpretation of many diseases including TED. In this respect, our attempt to use 3D VAE architecture was methodically the right choice.

Figure 4C shows that the model trained with normal CT images generated the extraocular muscles with a standard internal volume different from the abnormal CT images. The enlarged extraocular muscle in TED patients produces a larger difference between the input and output images; thus, we presume that our 3D VAE model identified abnormalities in abnormal CT images in a manner similar to the diagnostic approach used by physicians. However, some abnormal CT images correctly identified by the model showed large differences in extraorbital structures the structures. Figure 4D shows that the model initially distinguished the enlarged extraocular muscles in some input images from abnormal CT images. However, the total loss value did not exceed the best cut-off value, and these images were erroneously identified as normal. Thus, other factors (in addition to extraocular muscles) may have been associated with increased total losses.

These noisy image conditions are not confined to imaging studies and need to be overcome. One study tried data augmentation via synthetic data generating by using of 3D generative adversarial network (GAN) for better bioimage analysis^19^. The study showed a more realistic signal-to-background intensity ratio and improved accuracy. Another study proposed a VAE model that avoids noise introduced by the random selection of negative samples unlike previous models for the prediction of potential miRNA-disease association^20^. Its main mechanism is based on the two spliced matrices, which were applied to train the VAE respectively. The model showed that most of the potentially associated miRNAs were verified by databases. Like these two studies, if we develop our proposed model to decrease noise image conditions, model performance may be improved due to a decrease in the total loss associated with extraorbital structures. Future studies are needed to test this hypothesis.

In the otolaryngology field, a 3D VAE was used to detect inner ear abnormalities in CT images^21^. The model achieved a very high area under the ROC curve value of 0.99, and 99.1% sensitivity and 92.0% specificity, when using the colored pixel ratio to evaluate model performance. Notably, that study used 6,663 normal and 113 abnormal temporal bone CT images, respectively, for unsupervised DL. In our study, we applied several evaluation methods. However, we did not achieve performance above ROC curve analysis. As mentioned above, the size of the training dataset may have influenced model performance.

To our knowledge, no previous study has applied unsupervised DL to a small dataset of CT images. Most DL studies involving small numbers of medical images used supervised^22–24^, or semi-supervised DL^25–27^. These approaches may be used because supervised DL algorithms are expected to identify features that distinguish among data in small datasets; they require definite answers to focus on during the model training. Unsupervised DL also extracts and learns features from input data. However, in contrast to supervised DL, unsupervised DL is not provided which part to focus on exactly, thus seems to learn something different from what we intended. particularly in small datasets. In this study, we preprocessed input CT images and emphasized the extraocular muscles; this is because the feature extraction and image reconstruction in our 3D VAE model were mainly focused on orbital bone contours, which exhibited higher pixel values compared with extraocular muscles in the non-preprocessed original CT images. Thus, our model did not distinguish abnormal CT images with enlarged extraocular muscles from normal CT images. This process is a type of feature engineering and was possible because one of the authors is an ophthalmologic specialist which has understood the features of CT images. Previous studies also suggested that feature engineering based on domain knowledge may be a more suitable machine learning strategy with the notable advantages of the studies of medical images (e.g., CT or MRI) for analysis with small datasets^28,29^. Importantly, in our study superior performance was not achieved compared with DL studies involving larger datasets; more efficient DL can be performed when researchers have sufficient domain knowledge and apply it to DL, regardless of limitations in dataset size.

In conclusion, we proposed an unsupervised model that successfully detected abnormalities in small extraocular muscles using a small dataset, although it could not completely distinguish between normal and abnormal extraocular muscles. Unsupervised learning could serve as an alternative detection method for medical imaging studies in which annotation is difficult or impossible to perform.

## Methods

### Deep neural network architecture

Autoencoders (AEs) are fully developed in an unsupervised manner. Compression and decompression are automatically inferred from data, rather than using mathematical equations or hand-crafted features. Thus, no annotation information is required during AE training. AEs can be used to reduce data dimensionality^30^ and facilitate feature extraction. AEs consist of three main components: an encoder, a decoder, and a latent dimension module. The encoder compresses the input images, extracts features, and maps the features to a code in a latent dimension. The decoder reconstructs images from the code. The latent dimension is a representation of the dataset typically used for dimensionality reduction; this representation includes a smaller number of nodes and is generated by training input images to ignore unimportant data.

Variational autoencoders (VAEs) enable probabilistic descriptions of observation in latent dimensions^31^. VAEs enable feature encoding and decoding under limited conditions. Model-generated vectors are forced to conform to a Gaussian distribution to control image generation^32^. VAEs learn latent variables with continuous distributions, which are useful for generative modeling tasks. The encoder component produces a set of two vectors in each latent dimension: a vector of means (μ) and a vector of standard deviations (σ). Accordingly, VAEs attempt to learn the distributions of latent variables based on the mean values and their variances, rather than the deterministic mapping used by conventional AEs^31,33,34^. Compared with two-dimensional (2D) convolutional neural networks, a 3D convolutional neural network model can encode representations of volumetric fields and therefore extract more discriminative features via the 3D spatial information^17^. However, long computational times and a lack of pre-trained models are limitations of 3D convolutional neural network models.

**Figure 5:**
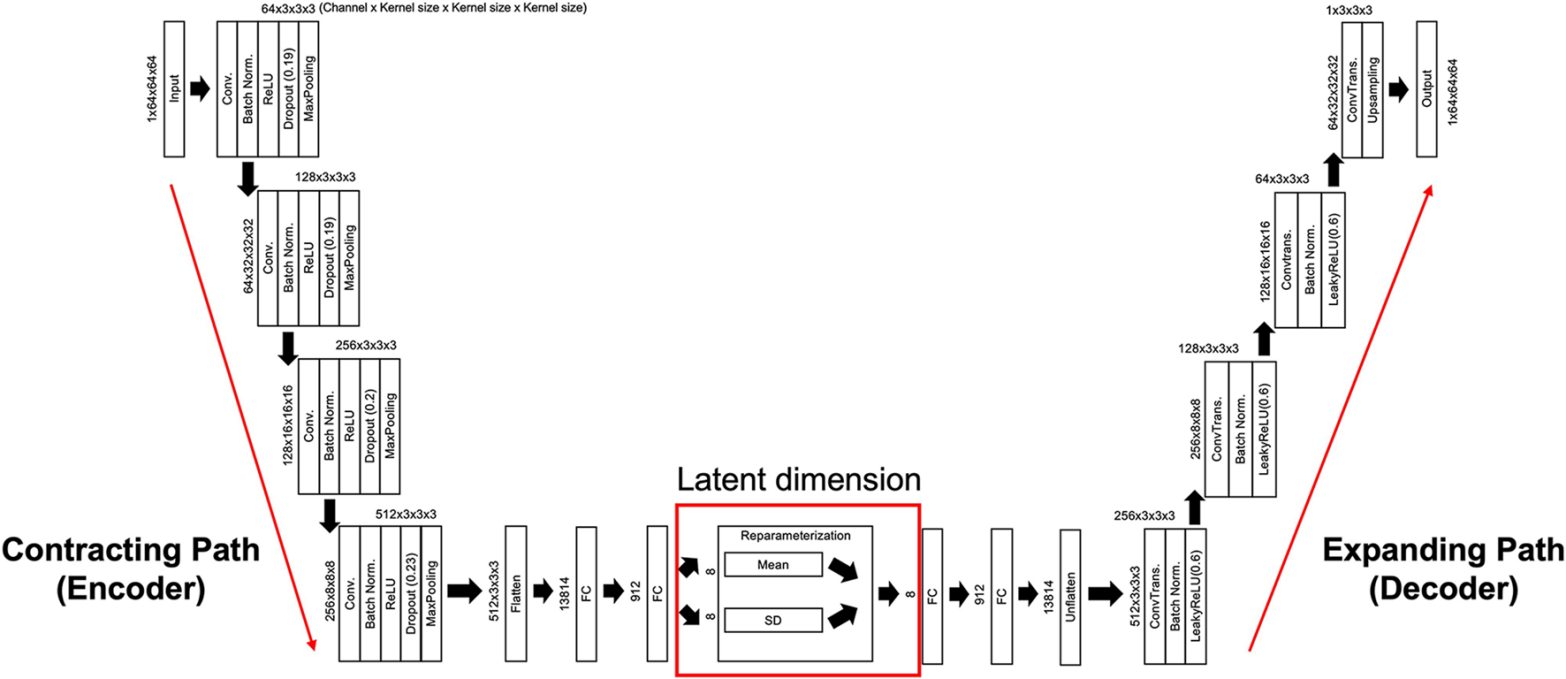
Schematic view of our proposed 3D VAE model, which consists of an encoder, latent dimension module, and decoder. Batch Norm., batch normalization; Conv, 3D convolution; ConvTrans, 3D transposed convolution; FC, fully connected; ReLU, rectified linear unit.

Our proposed 3D VAE model was constructed within the DL framework PyTorch. The encoder consisted of four modules. Each module had four successive layers: 3D convolution, batch normalization layer, rectified linear unit (ReLU) activation function, dropout, and 3D max pooling. The dropout layer was inserted into each module to reduce the initial overfitting of training data during training epochs, which is the first characteristic of our model that distinguishes it from conventional VAE models. Next, two fully connected layers were stacked into a hierarchical architecture. Based on vectors output from the encoder, eight latent vectors were calculated from latent variables in the reparameterization module. These latent vectors passed through fully connected layers, reorganized, and inserted into the decoder. The decoder consisted of three modules with three layers (3D transposed convolution, batch normalization, leaky ReLU activation function) and one module (a combination of 3D transposed convolution, and upsampling). The last module layer was composed of an upsampling layer, rather than a transposed convolutional layer, is the second characteristic of our model because the output produced through the proposed model reduced margin checkerboard artifacts. All data were resized to 64 × 64 × 64 voxels and input into the model (Fig. 5).

### Datasets

A retrospective review was performed to identify patients who underwent orbital CT scans at St. Vincent’s Hospital in South Korea from 2017 to 2022. According to radiologic specialists, 208 CT scans were considered normal and 54 showed signs of TED. All patients with signs of TED were subsequently diagnosed with TED according to abnormal thyroid function test results (T3, free T4, and thyroid-stimulating hormone) and the presence of thickening in at least one extraocular muscle. The study protocol was approved by the Institutional Review Board of our hospital (XC19REGI0076), and the study was performed in accordance with the Declaration of Helsinki. Informed consent was obtained from all subjects or their legal guardians.

### Preprocessing

**Figure 6:**
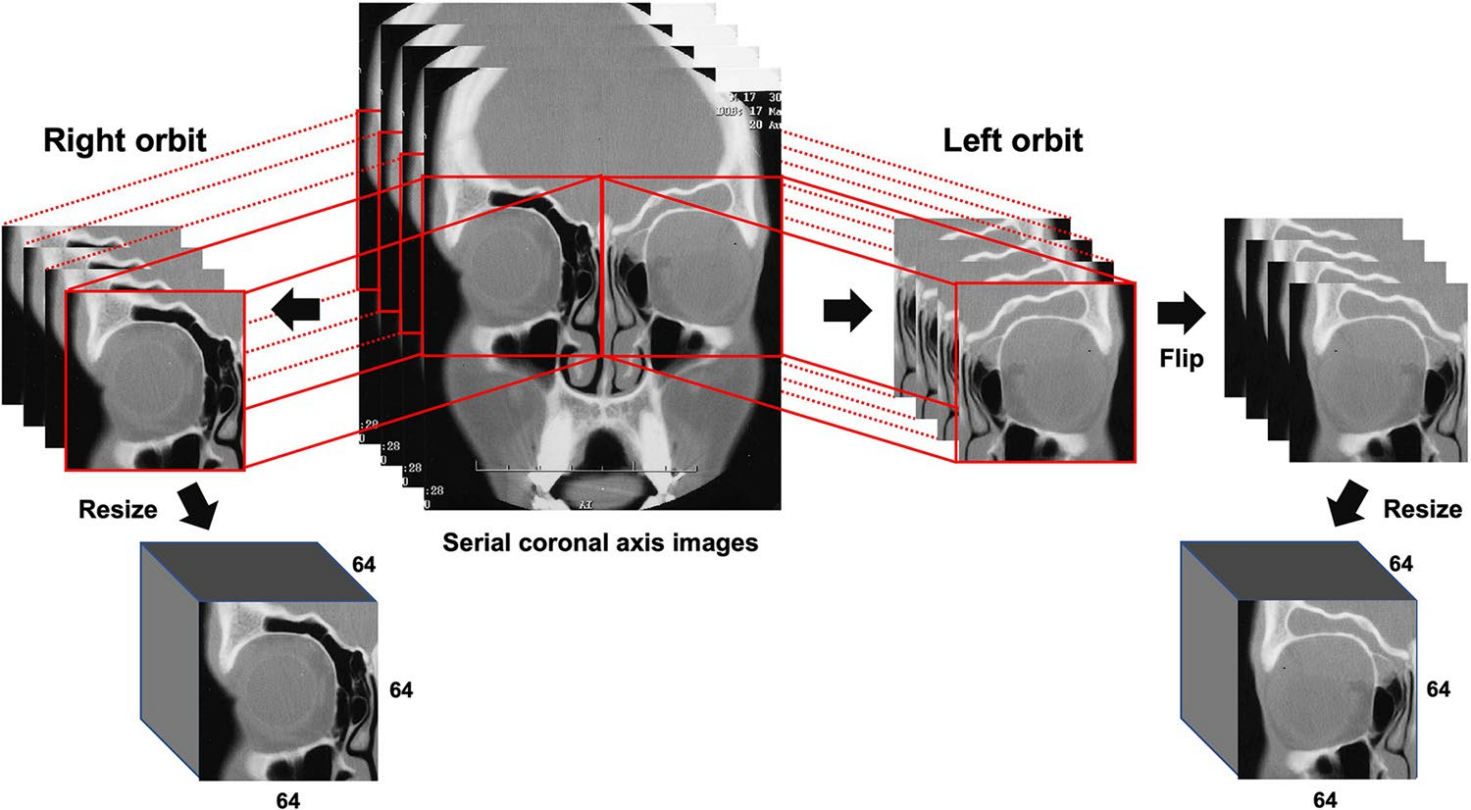
Preprocessing of CT images. Only coronal axis view orbital CT images were selected. Orbital areas were extracted, and each left orbit was flipped to match the shape of the right orbit images. Finally, the images in one CT were resized to 64 × 64 × 64 voxels with one channel.

**Figure 7:**
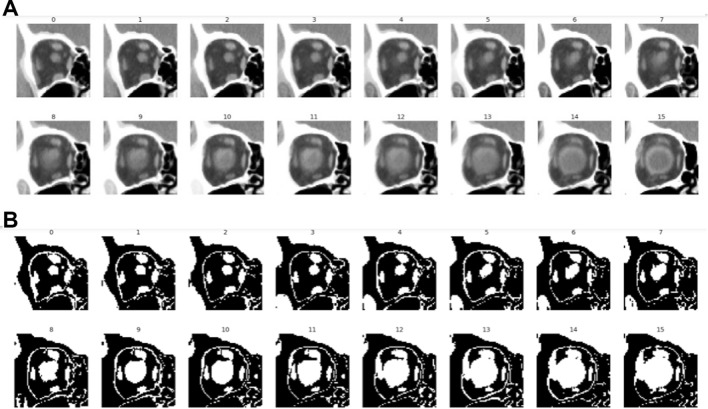
Representative partial CT image series (**A**) before and (**B**) after pixel value adjustments.

**Figure 8:**
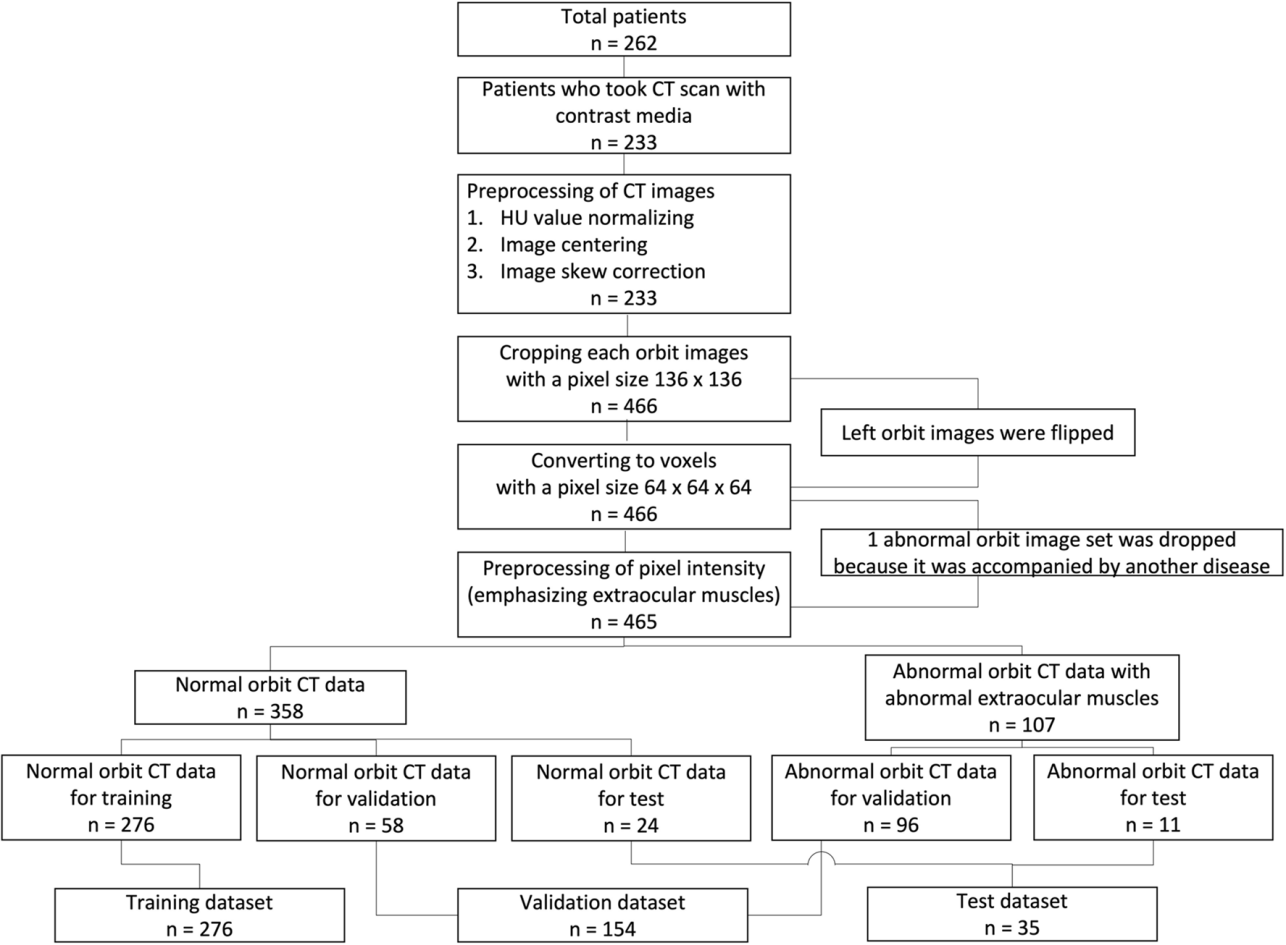
The sequential flow of preprocessing. *CT* Computed tomography, *HU* Hounsfield unit.

During model training, preprocessing was necessary to enhance model performance and reduce the negative effects of small datasets (relative to the large number of imaging parameters). Of the 262 CT scans collected, only 233 contrast-enhanced CT images in the coronal axis view were analyzed. The Hounsfield unit (HU) value of each pixel was adjusted for reading orbit CT images (window center; 40, window width; 400) and was normalized to the range of [0, 1] using min–max normalization for model training. All images were centered and subjected to skew correction. Each orbit was extracted from normal CT scans, with a pixel size of 136 × 136; in TED CT scans, only orbits with enlarged extraocular muscles were extracted. All extracted left orbit images were flipped to match the shape of the right orbit images. Finally, the extracted serial images of orbits were converted into 64 × 64 × 64 voxels with one channel (Fig. 6). The pixel values of input data were adjusted to emphasize extraocular muscles and suppress background noise, including high signal intensity from bones (which could interfere with model training for normal extraocular muscles; Fig. 7), which is the third characteristic of our model. Finally, 358 normal and 107 abnormal orbital CT images were prepared for model training, validation, and test datasets. Figure 8 shows the sequential flow of preprocessing.

### Training methods

In total, 276 normal orbital CT images were used for model training only (training dataset); 58 normal orbital CT images were used as the normal validation dataset (normal validation CT group). 96 TED orbital CT images were used as the abnormal validation dataset (abnormal validation CT group). The validation datasets were not used for model training, and the training datasets were not used for validation. Training data were input randomly (32 per training epoch), to avoid bias associated with the data being input in the same order during each training epoch. The dropout rate, the number of latent dimensions, and the negative slope of leaky ReLU were used as hyperparameters. After 100 epochs of training, normal and abnormal validation data were evaluated using trained parameters. We made a scatter plot using total loss values calculated through the model and evaluated whether normal and abnormal data could be distinguished. During model tuning, the best value for each hyperparameter was determined based on the following requirements: the training data did not exhibit overfitting during designated training epochs, and both normal and abnormal validation data exhibited stability in terms of total loss values with an increasing number of training epochs. The final hyperparameters were as follows. The dropout rate was 0.19 at the first 2 layers, 0.2 at the third layer, and 0.23 at the fourth layer. The number of latent dimensions was 8, and the negative slope of leaky ReLU was 0.6.

### Model verification: construction of the loss function

The loss function consisted of reproduction loss and Kullback–Leibler divergence (KLD) loss. Reproduction loss compared the model output and input images, and was calculated as the sum of squares of the difference in corresponding pixel locations between the input and output images. KLD describes how one probability distribution (i.e., input images) differs from another (i.e., output images). This loss function compares a latent vector with a zero mean, unit variance Gaussian distribution; the VAE is penalized if it begins to produce latent vectors that deviate from the desired distribution^35^. The reconstruction loss is intuitive because the negative log-likelihood of datapoints reflects the extent to which the VAE reconstructed the original data. The addition of KLD ensures that the generated latent vectors remain diverse, and prevents substantially different representations of similar data. This prevents the VAE from memorizing representations by plotting them in any area of a latent dimension^36^.

The loss function of the proposed model is defined as follows:

$$z=latent\,vector$$

$$x=input\,data$$

$$Encoder={q}_{\theta }\left(z{\vert}x\right)$$

$$Decoder= {p}_{\varnothing }\left(x{\vert}z\right)$$

$$p\left(z\right)=Normal(0, 1)$$

$$Loss=Reconstruction\,Loss+KLD\,loss$$

$$= {E}_{{q}_{\theta }\left(x{\vert}z\right)}[\mathrm{log}{p}_{\varnothing }(x{\vert}z)]-KLD({q}_{\theta }\left(z{\vert}x\right) \Vert p\left(z\right))$$

where the θ and ɸ represent the individual sets of weights adjusted for during training with the encoder and decoder, respectively.

To evaluate model performance, images in the normal validation CT group were labeled with 0, whereas while those in the abnormal CT group were labeled with 1. We performed receiver operating characteristic (ROC) curve analysis using the output total loss values from normal and abnormal CT groups and identified the optimal cut-off^37^.

### The difference map: visualization of differences between input and output images

Pixel values between each input and output images were squared pixel-wisely to emphasize differences between input and output images. The pixel position where there was a large difference was expressed in red, and the pixel position where there was a small difference was expressed in blue, like conventional heat map presentations.

## Supplementary Information

Supplementary Information 1.

## Data Availability

The implementation PyTorch codes and saved parameter weights generated during and/or analyzed during the current study are included in Supplementary Information files.
